# Description of a National Initiative to Train and Implement Processes of Primary Palliative Care in the Public Health System in Brazil

**DOI:** 10.1089/pmr.2024.0090

**Published:** 2025-03-17

**Authors:** Maria Perez Soares D’Alessandro, Leonardo Bohner Hoffmann, Hieda Ludugerio de Souza, Fábio Holanda Lacerda, Fernanda Spiel Tuoto, Ana Paula Mirarchi Vieira Maiello, Daniel Felgueiras Rolo, Taís Milene Santos de Paiva, Eliana Maria Ribeiro Dourado, Carina Tischler Pires, Vânia Bezerra, Daniel Neves Forte

**Affiliations:** ^1^ Sociedade Beneficente de Senhoras Hospital Sírio-Libanês, Diretoria de Compromisso Social, Sao Paulo, Brazil.; ^2^ Ministério da Saúde, Secretaria de Atenção Especializada à Saúde, Brasilia, Brazil.; ^3^ Conselho Nacional de Secretários de Saúde, Brasilia, Brazil.

**Keywords:** palliative care, public health, education, quality improvement

## Abstract

**Objective:**

To describe the first national project in Brazil to systematically bring palliative care (PC) knowledge and practices to public health services.

**Materials and Methods:**

Descriptive case study of “Palliative Care Program in the SUS—hospital care, specialized outpatient care and home care,” based on three main pillars and carried out in five stages.

**Results:**

The program took place from 2020 to 2024. Part of its fundamental design was the customization of PC initiatives to be implemented in the health care services based on the local reality of each region and its available resources. The target services were clusters of three public or philanthropic health care services comprising one hospital, one specialized outpatient clinic, and one home care service that allowed for the continual care of the patients.

**Discussion:**

Considering the Brazilian territorial extension and the complexity of the public health system, the project was innovative in adding competence and management processes in PC without burdening the services. Remote activities and reduced visits made the project low-cost, allowing it to be scaled up and viable even in a country of continental proportions.

**Conclusions:**

The field teams had the opportunity to listen to the health professionals of the public system, who were struggling with work overloads and a population in dire PC needs, giving them a voice and enabling the adjustment of the project’s interventions based on their reality. These ongoing and timely adjustments at the same time engaged local leaders and improved the interventions sought.

## Introduction

The level of palliative care (PC) development in Brazil is as heterogeneous and complex as the country itself, which hosts one of the largest universal free public health systems in the world—the Sistema Único de Saúde (SUS). While three-quarters of the population exclusively depend on the public health care system, only half of the PC services are public, and almost half of these are located in the southeast region of the country.^1^ Also, some regions are so precarious in resources that struggle to provide basic health care assistance, far from a PC ideal,^2^ which poses a great challenge taken by the project described hereby to develop PC.

The 2018 Lancet Palliative Care Commission Report recognizes an abyss between the need and the access for PC and pain relief in low and middle-income countries.^3^ The World Health Organization (WHO) recognizes that basic, intermediate, and specialty-level training are all needed to make basic PC services universally accessible to patients. However, despite WHO recommendations, many Latin American countries have an insufficient level of development in PC, including Brazil.^4,5^

In 2009, the Brazilian Ministry of Health (MoH) started a public–private partnership with philanthropic hospitals in order to support SUS development named “Programa de Apoio ao Desenvolvimento Institucional do SUS” (PROADI-SUS). This program has different projects related to the evaluation and incorporation of technologies, clinical research, specialized assistance, and management consultancy for health services improvement. The data or outcomes presented in this article were obtained from the project “Programa de Cuidados Paliativos no SUS—atenção hospitalar, ambulatorial especializada e atenção domiciliar (Program of Palliative Care in SUS—hospital care, specialized outpatient care and home care)” in partnership with the Brazilian MoH, through PROADI–SUS. The project was also developed with the support of the National Counsel of Health States Secretaries (Conass). Both MoH and Conass were the Governmental Representatives (GR) of the project.

In 2018, the first health resolution regarding PC in Brazil was published,^6^ a milestone in the process of making PC a health policy in the hope that it will culminate in a significant social impact, long-term wise. In practice, the resolution recognized the population need of PC, enabling initiatives such as this project to develop.

The project results chain consists of the following^7^: Inputs: a specialized team, training programs and a PC manual; Activities: running a PC diagnostics of the health care services, implementation of action plans, and training health care professionals; Outputs: PC protocols and processes established, and health care professionals trained in primary PC (PPC).

Thus, from 2020 to 2024, this PROADI-SUS project focused on developing PPC in services of the national public health care system in order to improve the quality of care offered to patients with life-threatening illness and their families. As such, the goal of this article is to describe this first national-level initiative to systematically take PC knowledge and practices throughout the SUS.

## Materials and Methods

This is a descriptive case study, conducted by the Sociedade Beneficente de Senhoras Hospital Sírio-Libanês (SBS-HSL), São Paulo, Brazil, within the scope of the PROADI-SUS initiative, through a partnership with the Brazilian MoH.

### The three main pillars of the project

The Program of Palliative Care in SUS was organized in three different but complementary actions: a diagnosis of the maturity level in PC in each health care service, implementation and improvement of the current processes and protocols in PC, training programs in PC for the health care professionals working in such institutions.

#### Diagnosis of the maturity level in PC

This pillar was based on an original assessment tool to identify the development level of PC in the services, evaluating six dimensions: (1) adequate resource allocation, (2) protocols and directives, (3) goals of care, (4) PC drug availability, (5) team and PC, and (6) health network integration. Each dimension generated a score from 0% to 100%, which, in turn, would give an overall PC development percentage rating.

All data were collected through four methods: (1) The service provided data via the Research Electronic Data Capture (REDCap) regarding the institution, its team of professionals, and the profile of the patients assisted; (2) a sample of the professionals was invited to anonymously provide information on the local culture regarding PC through REDCap questionnaire; (3) the trio of professionals working for the PROADI-SUS project visited each health care service twice in order to experience the local reality and later filled out a checklist of basic information; and (4) during these visits the trio gathered qualitative data on the current health care professionals’ understanding and the service’s culture in PC through focal groups.

The maturity score was complemented by a qualitative diagnosis of strengths and points for improvement regarding PC.

#### Implementation and improvement of processes in PPC

Some management processes targeted by the project included patient admission to the intensive care unit (ICU) based on the criteria established by the national board of medicine, called Conselho Federal de Medicina (CFM).^8^ In Brazil, triage criteria for ICU admission are needed due to the lack of ICU beds, especially in the public health system. The lack of ICU beds faced by some countries during COVID pandemic is unfortunately a long-lasting reality to most low and middle-income countries. Training and implementing this triage process for ICU referral aimed to improve the indication of adequate treatment to patients, decrease the prevalence of futile end-of-life interventions, and optimize public resources.

Another main goal was to increase the proportion of patients’ deaths with comfort measures documented on their medical record, as opposed to patients who received potentially inappropriate interventions on their last days of life. This was accomplished by standardizing the early application of PC needs identification tools and by discussing and documenting the goals of care in the medical record.

Furthermore, other specific plans of action were suggested in accordance with the local teams and local managers based on the context of each service, such as symptom management protocols, transitions of care protocols for discharging patients with PC needs to a community home care service, and burnout prevention strategies.

#### Training programs in PPC

The training program consisted of three different approaches.

The first one was an asynchronous, 20 credit-hours, long distance-course called “Cuidados Paliativos para Não-paliativistas” (Palliative Care for Non-palliatives), based on the Instituto de Ensino e Pesquisa (IEP) of SBS-HSL. Only health care professionals with university majors could apply for this course. It consisted of two different versions, one for physicians and another for nonphysicians. It addressed basic topics in PC. Two hundred and fifty licenses were distributed per group of three services, and the approval criterion was a score of 70% or more on the final test.

The workshop deepened some fundamental topics in PC and was structured in eight synchronous, two-hour sessions, half of which were online and the other half in-person classes, all of them facilitated by the SBS-HSL field team. There were 45 licenses available for each group of services, and the participants were required to have enrolled in the long-distance course described above. The approval criterion was participating in 75% or more of the classes. The workshop classes’ themes were PC needs identification and discussions on adequate measures, ethical and legal aspects of end-of-life care, health care professional’s self-care, symptom management and teamwork, communication, prognosis and advanced care planning, end-of-life care and palliative sedation, and bereavement and spirituality.

The third approach was open classes on specific topics, designed to access a larger number of professionals, including the ones without any university major degree. Some classes were in person and some online. They summed 12 hours of classes and included the following themes, based on the PC manual developed by the project^9^: definitions and principles of PC for licensed practical nurses, theoretical-practical workshop on hypodermoclysis (subcutaneous access), PC in the ICU, pediatric PC, and social pain in PC.

### Five-stage program

**FIG. 1.**
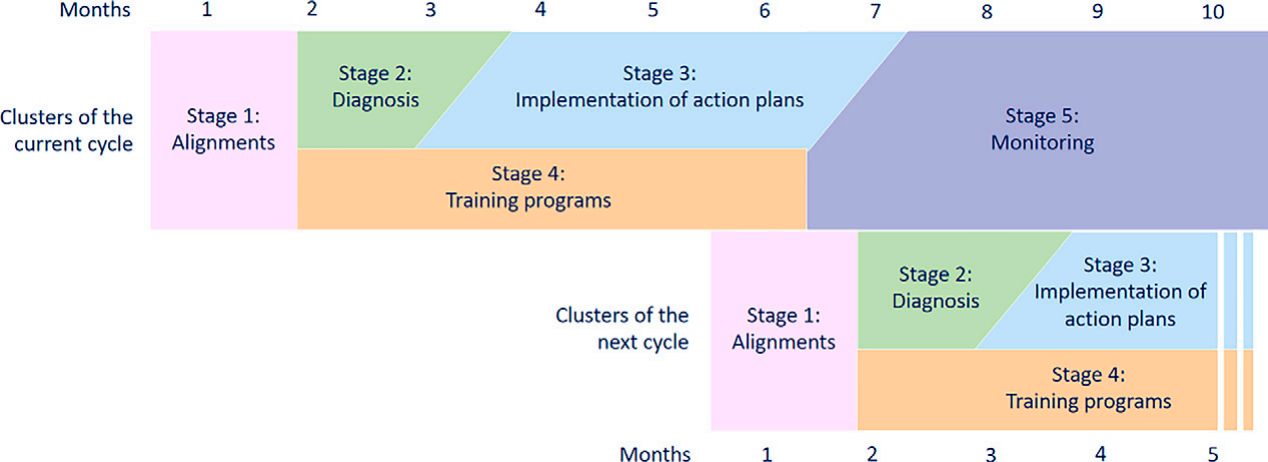
Schematics of the stages of the project.

The project was designed as a five-stage program over a period of 10 months (Fig. 1), in which clusters of services would participate at a time:

Stage 1: Alignment (1st month). In order to agree on which health care services would participate, there were discussions among the federal, state, and municipal spheres.

Stage 2: Diagnosis (2nd and 3rd months). The field team performed an analysis of the epidemiological profile of the services’ patients and assessed the maturity of their PC practices using the aforementioned tool and researched what the local culture of PC was.

Stage 3: Implementation of action plans (4th to 6th months). Based on the strengths and points for improvement identified on the diagnosis, along with a follow-up from the team of experts of the project, the local team decided on the best protocols and management processes to develop PPC according to the reality and possibilities of the service, with no added cost for the public health system. Examples of how this diagnosis oriented the action plans to be implemented are found in the results session.

Stage 4: Training programs (2nd to 6th months—this phase occurred concurrently with stages 2 and 3). This stage offered a long-distance course, a workshop, and other classes on specific themes, as well as a free PC manual developed by the same expert team, which considered the public health care system’s context with up-to-date content in Portuguese.^9^

Stage 5: Monitoring (7th to 10th months—this phase was concurrent with the first stages of the next cluster of services to receive the project). The teams followed up on the action plan indicators designed to evaluate the PC development by the health care services and teams in the different Brazilian regions. At last, the results of the project were presented at each locality.

## Results

From 2020 to 2024, SBS-HSL maintained the PROADI-SUS Program of Palliative Care. Part of its fundamental design was the customization of PC initiatives to be implemented in the health care services based on the local reality of each region and its available resources, as exemplified in Table 1. Moreover, no costs were added to the already burdened public services, allowing for a reorganization of the assistance provided at no extra costs. The main focus was to develop PPC approaches, based on the understanding that this is the best way to minimize the gap between the large scale of nationwide PC needs and the availability of specialized services.^4,10–13^ Therefore, the main objective of the project was the implementation and/or improvement of PC processes and training of health care professionals in order to benefit patients living with life-threatening diseases and their families. The target services were clusters of three public or philanthropic health care services in the same region aiming to build a PC network comprising one hospital, one specialized outpatient clinic, and one home care service that allowed for the continual care of the patients.

**Table 1.** Examples of Action Plans Based on the Diagnostics Run by the Assessment Tool

The six dimensions evaluated by the assessment tool	Example of action plan if the respective dimension needs improvement
Adequate resource allocation	Triage process for ICU referral
Protocols and directives	Clinical protocol of identification of patients with PC needs
Goals of care	Clinical protocol to define goals of care with the patient and the team
PC drug availability	Revision of availability of drugs for symptom management
Team and PC	Training of the team
Health network integration	Referral of the patients between hospital and home care service

ICU, intensive care unit; PC, palliative care.

### Project development

**FIG. 2.**
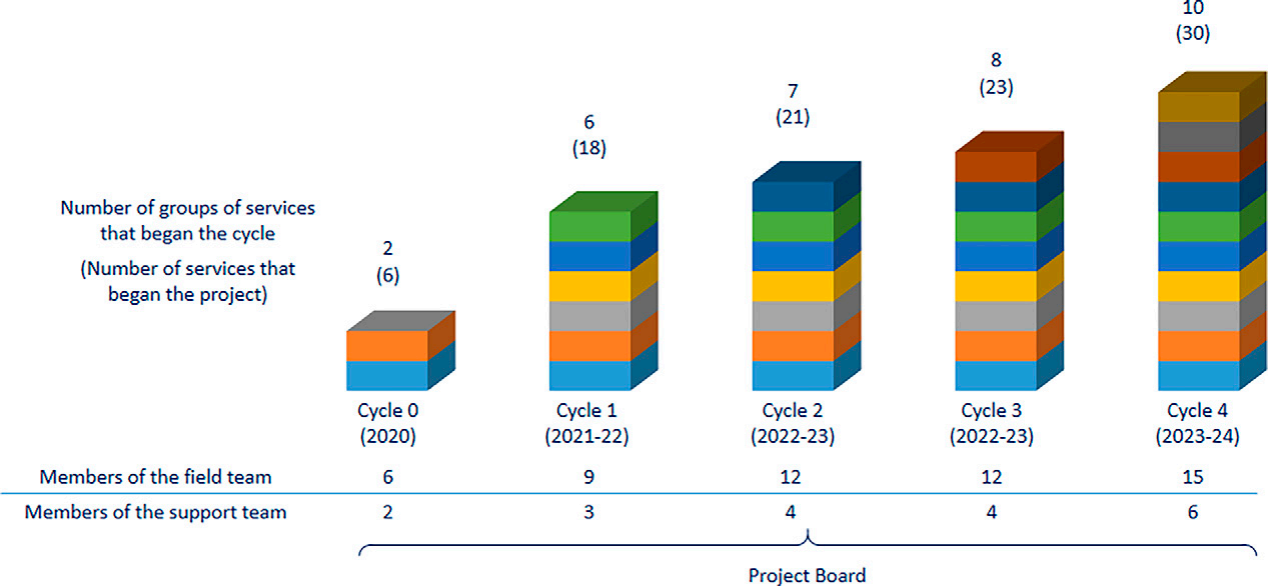
Growth of the project and its team.

The project grew modularly, with each new cycle involving more service clusters than the one before, a process that accompanied the increase of members on the team (Fig. 2). Most of the project’s professionals specialized in PC. The team was organized in three levels:

The field team was the most numerous because its role was to travel around Brazil visiting and working side by side with the services. Each trio of a physician, a psychologist, and a nurse was responsible for two different states with a cluster of three services each.

The support team acted as a mid-management, following up with the field teams and gathering and analyzing information and then reporting to the project managers. The support team was composed of a physician, a psychologist, a nurse, a social worker, an administrative assistant, and a data analyst.

The project board was composed of two project managers (a medical doctor and a project development specialist) and one medical advisor, whose purpose was to keep up with the rollout of the program and preserve its quality along its exponential growth. The board reported directly and periodically to the GR.

### Evaluation plan

A following study will take place to assess the outputs and outcomes of the project. The short-term results will be evaluated mainly through an indicator gathered since the baseline data and followed up to the conclusion of the project in the hospitals and home care services, which is the rate of comfort measures as a priority registered in the medical records of deceased patients. This performance indicator was chosen because it is objectively measurable, and it points to a better allocation of resources in end-of-life care as well as a better chance that patients with predictable death received comfort care. Also, we will gather data on training session attendance and participant demographics to evaluate engagement. Finally, we will survey the health care services to examine the implementation of PC needs identification tools.

## Discussion

Considering the territorial extent of Brazil and the complexity of the public health system at its various levels, the project presented itself as innovative by adding competence and implementing processes in PC without excessively burdening the health care services, since it considered each service’s local idiosyncrasies at the start and worked itself within each reality encountered. For instance, if a hospital had a specialized team, it was implemented a tool to identify PC needs at primary or specialized levels, in order to organize this assistance. Online activities allowed the potentiation and reduction of face-to-face visits, further reducing the program’s costs.

This descriptive study does not have the scope to present outcome results, which, though collected, are still being analyzed and will be published soon.

Challenges were encountered during the execution of the project, in particular the lack of support and dedication from some managers and medical staff in some regions, as well as the high professional turnover faced by other health care services. The project’s objectives in changing the PC culture in these places were hampered under these circumstances.

On the contrary, the major strengths of this project were the identification of local leaders associated with the local PC initiatives, as well as the customized and in-person implementation of the action plans designed to foster the culture in PPC throughout the country. The focus on PPC knowledge has also been perceived as an important tool in optimizing PC assistance where a specialized team already existed.

In this sense, the project management sought to fine-tune the selection criteria for the participating services in order to better take advantage of what this program had to offer and increase the chances of its perpetuity.

## Conclusions

This PPC program was implemented in Brazil. The field teams had the opportunity to listen to the health care professionals of the public system, who were struggling with work overloads and a population in dire PC needs, giving them a voice and enabling the adjustment of the project’s interventions based on their reality. These ongoing and timely adjustments at the same time engaged local leaders and improved the interventions sought. The MoH renewed and expanded the program up to 2026. Outcome results are being analyzed and will be published soon.

## Abbreviations Used

CFM: Conselho Federal de Medicina
Conass: Conselho Nacional de Secretários de Saúde
GR: Governmental Representatives
ICU: Intensive Care Unit
IEP: Instituto de Ensino e Pesquisa
MoH: Ministry of Health
PC: Palliative care
PPC: Primary palliative care
PROADI-SUS: Programa de Apoio ao Desenvolvimento Institucional do Sistema Único de Saúde
REDCap: Research Electronic Data Capture
SBS-HSL: Sociedade Beneficente de Senhoras Hospital Sírio-Libanês
SUS: Sistema Único de Saúde
WHO: World Health Organization
